# Recurrent plunging ranula

**DOI:** 10.4103/0971-9261.69143

**Published:** 2010

**Authors:** Pavai Arunachalam, Nithya Priyadharshini

**Affiliations:** Department of Paediatric Surgery, PSG Institute of Medical Science & Research, Peelamedu, Coimbatore, Tamil Nadu, India

**Keywords:** Plunging ranula, recurrence, salivary gland

## Abstract

We report two cases of plunging ranula, which had recurred after marsupialization. Both were successfully treated by removal of the ipsilateral sublingual gland. A brief review of the literature regarding the treatment options is presented.

## INTRODUCTION

Ranula is an extravasation cyst found in the floor of the mouth. They develop from extravasation of mucus after trauma to the sublingual gland or obstruction of the ducts.[1–3] Plunging ranulas are mucus retention cysts from sublingual gland or duct with extension into the submandibular space.[4] A variety of surgical procedures have been quoted in the literature ranging from marsupialization, excision of the ranula, sclerotherapy, and excision of the sublingual gland. The recurrence rate varies according to the procedure performed. These extravasation cysts originate from the sublingual salivary gland, and excision has been noted to be curative.

## CASE REPORTS

### Case 1

**Figure 1:**
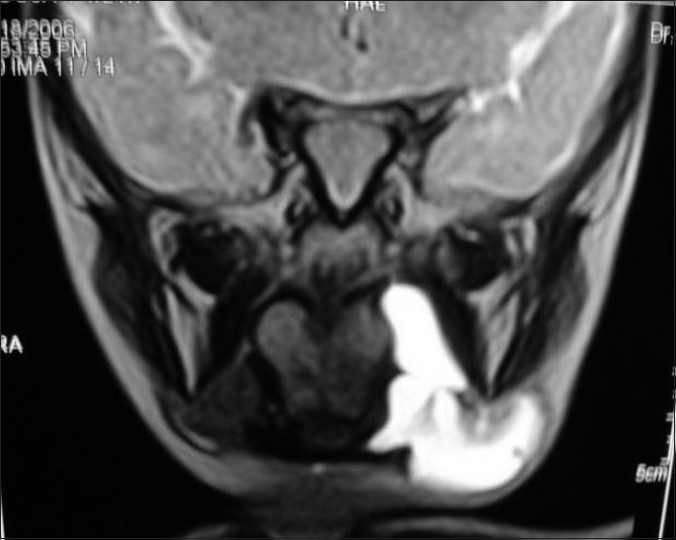
MRI showing cystic lesion on the left side in the sublingual region extending into the submental region, submandibular region, and the left parapharyngeal region up to the pharynx

An 8-month-old girl baby was referred with an intraoral swelling with extension into the submandibular and submental space. Marsupialization had been done at 2 months of age. A provisional diagnosis of plunging ranula was made. Magnetic resonance imaging (MRI) showed a thin walled cystic lesion on the left side involving the floor of the mouth in the sublingual region and extending inferiorly into the submental region, laterally into the submandibular region, superiorly into the left parapharyngeal region up to the pharynx [Figure 1]. Intraoral removal of the sublingual gland with drainage of the extravasation cyst was done. There was no recurrence after 4 years.

### Case 2

**Figure 2:**
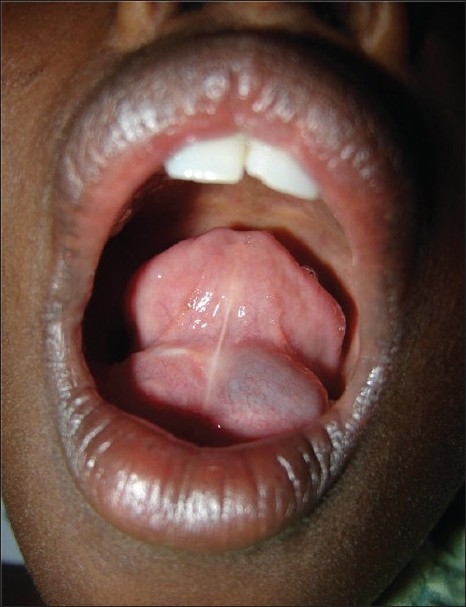
Cystic swelling in the floor of the mouth

A 10-year-old girl presented with a recurrent intraoral swelling. She had undergone marsupialization for an intraoral swelling 2 months earlier at a local hospital. The clinical examination revealed a large cystic swelling of 3 × 4 cm with extension into the submental space with cross-fluctuation [Figure 2]. The intraoral component had an extension to the opposite side. MRI showed a right-sided cystic swelling with extension to the opposite side and into the submental space. Excision of the right sublingual gland was done. There was no recurrence after 8 months.

## DISCUSSION

Ranula develops from extravasation of mucus after trauma to the sublingual gland or obstruction of the ducts.[2,3] Ranula can present at any age. It has been reported from 2 to 61 years of age with a slight female preponderance. The etiology is unknown, but it has been described in association with congenital anomalies, trauma, and disease of the sublingual gland.[5]

The pathophysiology involved in extravasation is hypertension in the duct due to obstruction leading to acinar rupture in the salivary gland and then extravasation of the mucus. The initial stage is a traumatic rupture of the excretory duct and the second stage is the extravasation and subsequent accumulation of saliva within the tissue, as shown by experimental studies.[6,7] Ranula in infancy may be due to duct agenesis, hypoplasia, or due to birth trauma. These extravasation cysts, which involve the sublingual salivary gland, can be classified into oral, mixed, or plunging and cervical ranula.

When these extravasation cysts extend into the submandibular or submental space, they are called plunging ranula. Its true incidence is unknown. The cause of plunging ranula is not known, but anomalies or obstruction of the salivary gland duct and naturally occurring defects in the mylohyoid muscle have been shown to be prerequisites for the extravasation. These cysts commonly extend into the submandibular triangle, occasionally they may extend superiorly into the parapharyngeal space as far as the base of the skull. They may extend inferiorly to the supraclavicular area and upper mediastinum or posteriorly into the retropharyngeal space.[5,8,9]

Ranula is a clinical diagnosis, and imaging studies are done mainly to know the extension of swelling prior to surgery or when the diagnosis is unclear. Computed tomography and specifically the presence of “tail sign” is pathognomonic for the plunging ranula.[10–12] This tail is due to extension behind the mylohyoid muscle and confirms the ranula to arise from the sublingual gland and is especially useful in differential diagnosis of cervical ranula. Aspiration cytology will show mucin with muciphages and biochemical analysis will show increase in amylase and protein content. This is diagnostic of the salivary origin.

Surgery is the main stay for the management of ranulas. These include incision and drainage, excision of ranula, marsupialization, and marsupialization with packing or complete excision of the sublingual gland. Simple marsupialization has fallen into disfavor primarily because of the failure rate, which has been anywhere from 61% to 89%.[1] Marsupialization with packing of the cyst cavity may reduce the recurrence. Takagi *et al*[4] have treated four cases with fenestration and continuous pressure with no recurrence. The only shortcoming of this is the necessity to keep the penrose drain for 3 weeks and the continuous compression. Hibernal *et al*[13] suggested that marsupialization should first be tried in pediatric population, but Yucca *et al*[14] had two recurrences in their nine cases and stated that excision of sublingual gland be reserved for recurrent ranulas. Crystal *et al*[1] have reported that the recurrence rate was 100% with incision and drainage, 61% in cases of simple marsupialization, and no recurrence in the case of excision of ranula with or without sublingual gland excision. In another study where 415 patients were followed-up, there was a recurrence of 66.6% after marsupialization, 57.69% after excision of ranula, and 1.2% after excision of sublingual gland.[15] Mahadevan *et al*[16] did not have a single case of recurrence after intraoral removal of the sublingual gland in their series of 21 pediatric patients. Extraoral excision has also been done due to the fear of injury to Wharton’s duct and lingual nerve. A review of surgical management of this lesion stresses the necessity of removal of the sublingual gland, which is the source of mucus formation rather than excision of the cyst. This will prevent recurrence of ranulas and occurrence of plunging ranula.

Other treatment modalities have also been utilized. Sclerotherapy with OK-432 is a good substitute for surgery.[17] Recurrence was noted in 14.3% and the patient had an average of 1.7 injections. Fukase *et al*[18] used a higher concentration in partially regressed cases and had 100% cure rate. Anecdotal reports of antenatal detection are present in the literature. Pires *et al*[19] have performed antenatal aspiration for decompression and stated that Ex-utero intra partum (EXIT) procedure may be useful in large swellings.

In conclusion, removal of the ipsilateral salivary gland is the management of choice in ranulas. Intralesional sclerotherapy with OK-432 is a good option but may not be feasible due to nonavailability of the product universally.
